# Staged cardiovascular magnetic resonance for differential diagnosis of Troponin T positive patients with low likelihood for acute coronary syndrome

**DOI:** 10.1186/1532-429X-12-51

**Published:** 2010-09-14

**Authors:** Henning Steen, Media Madadi-Schroeder, Stephanie Lehrke, Dirk Lossnitzer, Evangelos Giannitsis, Hugo A Katus

**Affiliations:** 1 Abteilung Innere Medizin III, Medizinische Klinik, Universitätsklinikum Heidelberg, Heidelberg, Germany

## Abstract

**Background:**

Cardiac Troponin-T (cTnT) is a cardio-specific indicator of myocardial necrosis due to ischemic or non-ischemic events. Considering the multiple causes of myocardial injury and treatment consequences there is great clinical need to clarify the underlying reason for cTnT release. We sought to implement acute CMR as a non-invasive imaging method for differential diagnosis of elevated cTnT in chest-pain unit (CPU) patients with non-conclusive symptoms and ECG-changes and a low to intermediate probability for coronary artery disease (CAD).

**Results:**

CPU patients (n = 29) who had positive cTnT were scanned at 1.5T with a new step-by-step CMR algorithm including cine-, perfusion-, T2-, angiography-and late gadolinium enhancement (LGE) imaging. For comparison patients also underwent echocardiography and coronary angiography if necessary. CMR was conducted successfully in all patients and detected 93% of cTnT releases of unknown cause, without adverse hemodynamic or arrhythmic events. Acute myocardial infarction was detected in 11, pulmonary embolism in 6, myocarditis in 5, renal disease and cardiomyopathy in 2, storage disorder in 1 patient. In 2 patients CMR was unable to reveal the cause of cTnT elevations. Mean CMR scan-time was 35 ± 8 min. In 4 patients, CMR led to immediate coronary angiography with correct prediction of the infarct related artery.

**Conclusions:**

We implemented a novel CMR algorithm to show the clinical value and practical feasibility of acute CMR in a non-conclusive patient cohort with unclear cTnT elevation. Since this pilot study has shown the feasibility of CMR in CPU patients, further prospective studies are warranted to compare CMR with other imaging modalities.

## Background

Cardiac troponin T (cTnT) is well established as preferred biomarker for detection of myocardial necrosis due to its absolute cardiospecificity [1]. Numerous clinical trials have established the role of cTnT in patients with suspected acute coronary syndrome for the diagnosis of acute myocardial infarction (AMI) as well as its power for risk strtification of patients with acute coronary syndromes (ACS) with [2] and without ST-segment elevation (STEMI/NSTEMI) [3].

Although the detection of cTnT in blood is specific for myocardial injury, it is not specific as to the cause of the myocardial damage. cTnT is also elevated in non-coronary cardiac diseases [4] including acute pulmonary embolism [5,6] (PE), acute heart failure [4], myocarditis [7] and toxic injury. If elevated in non-ACS conditions, cTnT elevations are associated with a high cardiac event rate, as shown for example in patients with end-stage renal disease (ESRD) [8].

In all these conditions cTnT elevations are associated with an adverse prognosis with a subsequent substantial need to diagnose and treat the underlying cause of cTnT liberation.

Cardiovascular magnetic resonance (CMR) is a non-invasive comprehensive imaging technique that simultaneously allows assessment of cardiac anatomy, tissue characterization and functional analysis of right and left ventricles (RV, LV). Cardiac dimensions, hypertrophy patterns as well as wall motion abnormalities can be easily visualized in breath-hold cine SSFP sequences with superior image quality [9]. Inflammation [10], myocardial hypo-perfusion [11] and infarct-related necrosis [12] are distinctly detectable using T2-edema-or late gadolinium enhancement (LGE) imaging techniques [13]. Furthermore, gadolinium can be utilized for high resolution pulmonary and aortic angiographies to exclude pulmonary embolism [14] or aortic dissection [15].

We sought to implement acute CMR and a novel step-by-step algorithm as a non-invasive diagnostic imaging method for differential diagnosis of elevated cTnT in hemo-dynamically stable patients with non-conclusive symptoms, non-diagnostic ECG, and low to intermediate probability for CAD [16].

## Methods

### Patients

Patients with an elevated cTnT (> 0.03 μg/L) had to fulfil the following criteria for inclusion: 1. low suspicion of ACS and 2. a) one potential differential diagnosis due to clinical symptoms, or b) certain laboratory findings (c-reactive protein elevation, elevated Wells score [17]). A low likelihood of ACS was defined as a) lack of typical angina, b) low-intermediate probability of CAD (≥2 cardiovascular risk factors, absent history of CAD) and c) normal/non-diagnostic ECG. Patients were consecutively enrolled in our CPU between January and June 2006. Patients with a high likelihood for ACS were excluded and received standard treatment and diagnostic work-up, such as immediate, early or deferred coronary angiography with or without coronary intervention. AMI was diagnosed using the ESC/AHA/ACC Federation Task Force redefinition of myocardial infarction guidelines [18]. Briefly, AMI required the detection of a rising or falling pattern of cTnT with at least one of the following: symptoms of ischemia, new ST-T changes on ECG, development of Q waves on ECG, or imaging evidence of new loss of viable myocardium. We excluded asymptomatic patients with ESRD and a constant level of cTnT in two consecutive samples obtained after an interval of at least 6 hours. In ESRD patients, a cTnT-change of ≥20% 6-9 h after presentation has been recommended to indicate an acute condition [19]. cTnT was regarded to be with a recommended diagnostic threshold of 0.03 μg/l. The study protocol was in accordance with the Declaration of Helsinki and approved by the local ethical committee. All patients included in the study gave informed written consent.

### Study protocol

After CPU admission and fulfilment of the CMR inclusion criteria, the study patients were consecutively asked and included. We excluded patients with severe dyspnoea, claustrophobia, implanted pacemaker/defibrillators or other metal devices. Patients who were admitted between 7pm and 8am were excluded since they were admitted outside the operating CMR hours. Patients were transported to the nearby CMR unit (~40 m distance) with a specific MRI trolley-system (Philips PhysioTrak, Best, the Netherlands) that provides the possibility of monitoring blood pressure, heart rate and blood oxygen saturation. The patients' medication, if necessary, was continued during transport and CMR using MR compatible infusion pumps.

During CMR scanning, one technician (B.H., A.W.), one doctor (H.S., S.L., D.L.) and one study assistant (M.M.) were present and analysed the images simultaneously. After diagnosis was made, CMR was stopped and the patient re-transported to the CPU.

Before or after CMR an additional echocardiography exam was accomplished in all patients for verification and comparison with CMR results. Post CMR, suspected ACS or non-coronary differential diagnoses were confirmed by either coronary angiography or 64-slice CT. When after CMR and echo the diagnosis was clear, invasive procedures were not obligatory to minimize patients' X-ray exposure.

### The eight-step CMR algorithm

**Figure 1:**
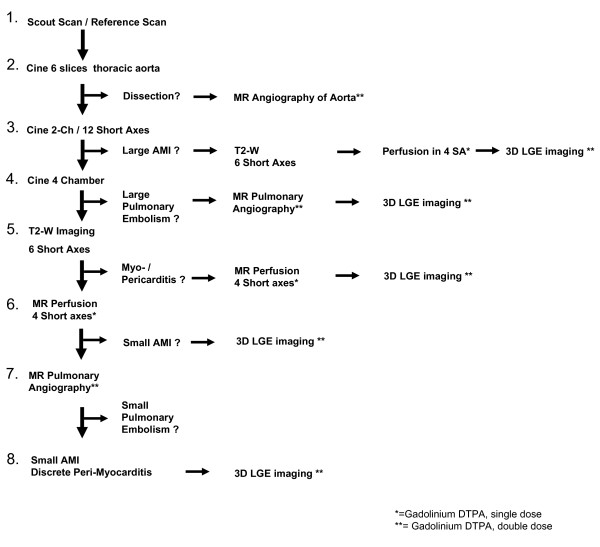
**Eight-step Heidelberg CMR algorithm**.

CMR was performed on a 1.5 T whole body CMR scanner (Achieva^®^, Philips Medical Systems, Best, the Netherlands). The pre-specified algorithm for stepwise evaluation is shown in figure 1. The algorithm could be followed step-by-step or interrupted at each point to minimize study scan time for the patient.

After scout and reference scans (*Step-1*), in *Step-2 *six SSFP-cine images with parallel imaging (slice-thickness = 8 mm; sense-factor = 2) were acquired through the thoracic aorta with subsequent MR angiography if dissection was suspected.

At *Step-3*, two-chamber and multiple short axis SSFP-cine images were scanned for wall motion abnormalities and edema [20] to rule out large AMI. If hypo-or akinesia was present with suspicion of edema, breath-hold T2 black-blood imaging (TSE-SPIR; slice-thickness = 8 mm; Sense-factor = 2.3) and gadolinium three-slice perfusion (Magnevist, Schering Germany;0.05 mmol/kg-bw) imaging (b-FFE; slice thickness = 8 mm;sense-factor = 2) were conducted. Ten minutes after additional 0.15 mmol/kg-bw gadolinium, multi-slice breath-hold LGE imaging (FFE-T1, TR/TE 2.6/0.9 ms, slice-thickness = 5 mm, rec. voxel 2/2/5 mm, sense-factor = 2, TI-time = 200-300 ms) followed to estimate infarct size and identify the affected coronary territory. CMR results were discussed with the interventional cardiologist for the need of catheterization.

*Step-4 *contained diagnosis of large PE by performing four-chamber SSFP-cine images to exclude wall motion abnormalities of the RV. If PE was suspected, a non-ECG-triggered 3D-MR pulmonary angiography (3D-FFE, halfscan-data acquisition; rec voxel = 0.7/0.7/2 mm;sense-factor = 2.2) was accomplished using double dose gadolinium.

After 10 minutes, LGE imaging was conducted to potentially visualize RV myocardial necrosis and pulmonary thrombi.

After triple-rule-out (*Step1-4*), *in Step-5 *T2-weighted short axes were imaged to rule-out edema in peri-/myocarditis followed by LGE imaging (see above) to detect patho-gnomonic inflammation patterns.

If no inflammation was seen, *Step-6 *contained multi-slice short axis perfusion imaging to detect small AMI and, in case of a perfusion defect, LGE imaging.

*Step-7 *looked for small PE performing 3D-MR pulmonary angiography, whereas ten minutes after gadolinium administration *Step-8 *served as tool for detection of small AMI or peri-/myocarditis. The time points for patient transportation, CMR preparation and scan duration were recorded.

### Statistics

Data were expressed as mean ± standard deviation or as median with inter-quartile range (IQR; 25-75th percentile) if not normally distributed. Differences between two groups were compared by Student's t-test or Mann-Whitney-U-Test for non-parametrical continuous variables. Continuous variables among more than 2 groups were compared by one-way ANOVA. Categorical variables between groups were compared by chi-square test or Fisher's exact test. For all analyses, p-values < 0.05 were regarded statistically significant. All statistical analyses were carried out using MedCalc 9.4.1.0 (MedCalc Statistical Software bvba, Belgium).

## Results

### Study screening and patient characteristics

Between January and July 2006, 1412 cTnT positive patients were admitted to our CPU. Out of 1,412 eligible patients during this time, 804 were admitted between 7pm and 8am (outside the operating times) and could therefore not be included. Of the remaining 608 patients, 110 had a STEMI and received interventional treatment. Of the now remaining 498 patients, 407 patients were clearly diagnosed with NSTEMI with either immediate or delayed invasive diagnostic treatments. Out of the rest of 91 patients, 55 showed either a unequivocal clinical signs of pulmonary embolism, myocarditis or renal failure without cTnT serum level changes cTnT-change of ≥20% 6-9 h after presentation. We were thus left with 36 patients who could potentially be included in this pilot study after careful investigations in the CPU. In seven of these 36 patients CMR could not be performed due to claustrophobia or metal implants (4/3 patients). Therefore, 29 patients were included for the final study. The characteristics of the study cohort are shown in table 1.

**Table 1:** Patient demographics and characteristics

No. of patients	29
Age (yrs)	57 ± 17
Sex f/m-n (%)	9/20 (31/69)
BMI (kg/m^2^)	25.6 ± 4.7
RR syst. (mmHg)	133 ± 9
RR diast. (mmHg)	77 ± 7
Heart rate (beats/min)	83 ± 19
Fever >37.5°C	6/29
Cardiovascular risk factors	
Diabetes - n (%)	6 (21)
Smoking -n (%)	12 (42)
Hypertension - n (%)	16 (55)
Hyperlipidemia-n (%)	13 (45)
Family history-n (%)	13 (21)
TIMI Score	2.2 ± 1.2
Wells Score	1.0 ± 1.4
Serum parameters	
cTnT admission (ng/dl)	0.9 ± 1.9
cTnT max. (ng/dl)	1.5 ± 3.9
Creatinine (mg/dl)	1.2 ± 0.6
GFR (ml/min)	75 ± 29
Leucocytes (/nl)	10.0 ± 4.3
...CRP (mg/dl)	45 ± 59
**ECG criteria**	
Sinus rhythm -n (%)	26 (90)
Tachycardia -n (%)	7 (24)
Left bundle branch block -n (%)	1 (3)
Right bundle branch block -n (%)	1 (3)
S1Q3 type -n (%)	1 (3)
T-inversion -n (%)	13 (45)
ST-deviation -n (%)	7 (24)

### Differential Diagnoses detected by CMR

**Figure 2:**
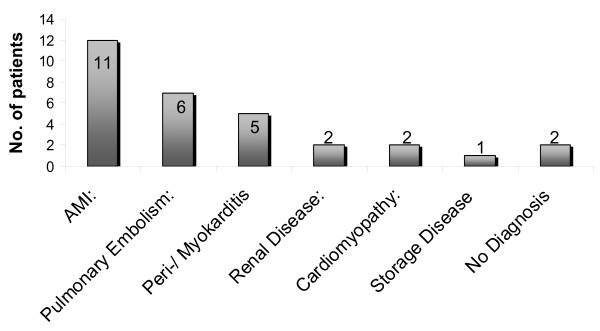
**Although clinically in-conclusive the study group comprised 11 AMI-, 6 PE-, 5 peri-/myocarditis-, 2 ESRD and CMP-and one amyloidosis patient**. In two patients no diagnosis could be found.

**Figure 3:**
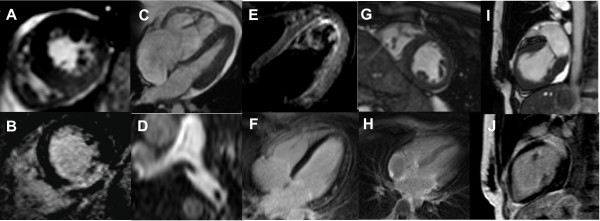
**A+B) 63 year-old patient with inferior wall AMI, T2-edema and hypo-kinesia on short- axis SSFP-images (A) and concomitant LGE (B)**. **C+D) **29 year-old patient with excessively dilated right chambers and systolic dysfunction on SSFP-images **(C) **revealing thrombi in the proximal left pulmonary artery **(D) **on pulmonary angiography. On echocardiography the patient showed only insufficient image quality. **E+F) **45 year-old patient with intermitting fever and edema at the LV lateral wall on T2-four chamber images **(E) **without wall motion abnormalities but clear patchy, infarct-atypical LGE images confirming the diagnosis of myocarditis **(F)**. **G+H) **32 year-old patient with slightly elevated cTnT levels, moderately reduced ejection fraction (EF = 43%), symmetric myocardial hypertrophy **(G) **and diffuse LGE patterns **(H) **suspicious of cardiac amyloidosis. **I+J) **62 year-old woman with signs of mid-ventricular ballooning **(I) **without edema or LGE **(J) **was classified as tako-tsubo CMP.

The CMR protocol was feasible in all 29 cases. During transportation, CMR preparation and scanning, no arrhythmias or hemo-dynamic instability were noted. In our study, the CMR diagnoses were AMI in 11, pulmonary embolism (PE) in 6, peri-/myocarditis in 5, elevated cTnT due to ESRD in 2, cardiomyopathy (CMP) in two and storage disease in 1 patient. In two patients, no diagnosis was found (figure 2). Some clinical CMR examples are given in figure 3.

AMI patients showed significantly more cardiovascular risk factors (2.9 ± 0.4, p = 0.04), higher admission cTnT (1.8 ± 3 ng/ml) and the highest TIMI score (2.6 ± 1.3) of all groups. CMR detected NSTEMI in nine of eleven cases. In coronary angiography, significant CAD ( > 50% luminal obstruction) could be ruled out in one case. Seven cases showed a single-vessel and three showed a two-vessel disease. Nine cases received PCI in at least one lesion.

In six patients PE was detected by CMR. Admission cTnT was significantly lower (0.15 ± 0.14 ng/ml;p = 0.023) compared to the AMI group, but had the highest Wells score^17 ^(2.2 ± 2). Since CMR clearly showed PE in four of six patients, only two patients received non-invasive CT for diagnosis confirmation. No heart catheterisation was demanded by the attending physician so that CMR saved invasive procedures in all six cases.

Five significantly younger patients (48 ± 32years;p < 0.05) with low incidence for cardiovascular risk factors (1.0 ± 0.1), low Wells (0.3 ± 0.7) but high serum levels of CRP (102 ± 81 mg/dl) revealed peri-/myocarditis on CMR.

Two patients with ESRD (creatine = 2.6 ± 0.1 mg/dl; MDRD = 31 ± 0 ml/min), known CAD and T-wave inversions with ST-deviations, but new onset of atypical chest pain, neither showed wall motion abnormality, edema, perfusion defect nor infarct-typical LGE on CMR. Therefore in both cases no coronary angiography was demanded by the attending physician.

Two elderly female patients without any cardiovascular risk factors, low Wells scores (0.5 ± 0.3) and moderate cTnT levels (0.28 μg/l ± 0.1) revealed either tachy-arrhythmia or ST-deviation after psychogenic stress. CMR showed apical ballooning without T2-edema or LGE and classified them as Tako-Tsubo cardiomyopathy.

One younger patient with a moderate cTnT level (0.31 ng/ml), three CAD risk factors, sinus-tachycardia and T-wave inversions showed a moderately reduced ejection fraction (EF = 43%) and symmetric myocardial hypertrophy with thickened heart valves. Surprisingly, LGE imaging showed amyloidosis gadolinium-patterns. The patient refused myocardial biopsy but coronary angiography showed an insignificant LAD lesion. The patient refused any further diagnostics and discharged himself prematurely.

In two elderly patients with low cardiovascular risk profile (0.5 ± 0.5), marginally elevated cTnT (0.03 μg/l), good renal function (1.2 ± 01 mg/dl, 96 ± 42 ml/min), inconspicuous lab-works, no high grade stenosis on X-ray but new arrhythmia with T-wave inversions, the underlying reason for the cTnT elevation could not be identified.

### Comparison of diagnostic methods

In nine of eleven AMI patients, CMR made the correct diagnosis. In one of them cTnT was only minimally elevated (0.05 ng/ml) with incompliance of breath-hold commands. The other patient showed tachy-arrhythmia with consecutive blurred imaging. Both patients were also misdiagnosed by echocardiography. However, when compared to CMR, echocardiography missed another four AMI patients due to not detected wall motion abnormalities or insufficient acoustic windows. In four cases, patients with CMR diagnosed NSTEMI received an earlier coronary angiography procedure due to large CMR perfusion defects and pronounced LGE patterns. In all AMI cases, CMR predicted the affected coronary vessel correctly. Moreover, CMR distinguished all six patients with PE, whereas echocardiography missed half of them due to insufficient image quality. Since CMR showed the exact localisation of the pulmonary thrombi as well as right ventricular function in at least four of six patients undoubtedly, coronary angiography was not conducted.

Similarly, CMR distinguished conclusively all patients with peri-/myocarditis because of characteristic gadolinium patterns. In only one case with regional wall motion abnormalities and pericardial effusion, echocardiography offered the correct diagnosis. Again, CMR saved coronary angiography procedures in these patients. In two patients, where all three methods could not find a diagnosis, CMR could at least rule out wall motion abnormalities, perfusion defects, edema or significant myocardial necrosis in all 17 AHA segments, which could not be accomplished by the two other methods. In the two cases of renal dysfunction, CMR again could not show any acute pathology, so that decisions could be made not to proceed with coronary angiography since the coronary status was known from former exams.

### CMR time course for differential diagnoses

**Figure 4:**
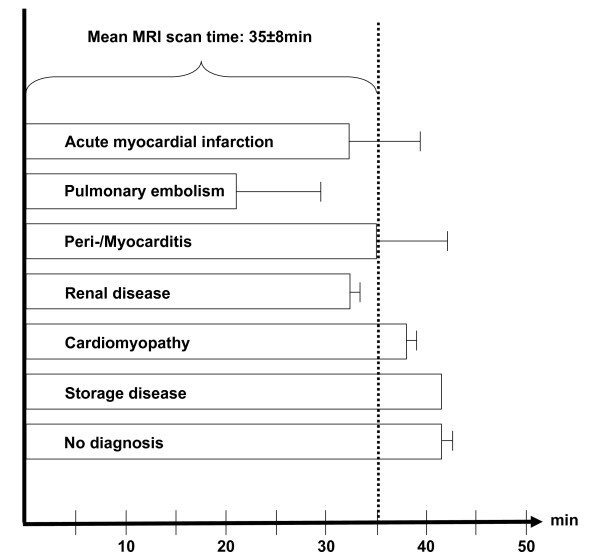
**Time bars with average CMR time durations for all differential diagnoses**.

In figure 4 seven time bars with average duration of CMR in the different groups are presented. The overall time duration was approximately 1 hour. Mean transportation time to the CMR scanner was 2.5 ± 1 min, preparation time 15 ± 2 min, CMR scan time was between 24 ± 9 min for PE and 44 ± 1 min for patients in which no diagnosis could be found. Transportation back to the CPU after CMR was 11 ± 3 min.

## Discussion

For the first time, we show in this prospective pilot study that CMR is useful for confirmation of ACS, adds information on infarct localisation and extent, and may provide a conclusive differential diagnosis in the majority of patients with elevated cTnT concentrations but equivocal signs or symptoms of ACS. Thus, CMR may improve the diagnostic work-up, and may be helpful for patient management. CMR can be completed within 35 minutes, is safe and well tolerated.

### CMR for the differential diagnosis of elevated cTnT in patients with low or intermediate probability of ACS

CTnT is highly sensitive and characterized by absolute tissue specificity for myocardium. As elevated cTnT is not always due to myocardial infarction, cTnT in patients with equivocal signs or symptoms for ACS may be caused by differential diagnoses including myocarditis [13], PE [14] or ESRD [19]. Accordingly, confirmation of suspected ACS or accurate differential diagnosis is of paramount importance to avoid unnecessary invasive procedures and to customize therapies. Although the diagnosis of ACS was equivocal in our patients, 30% had cTnT elevation due to NSTEMI and 9 of 11 cases required subsequent PCI. And although the Wells score demonstrated low clinical pre-test probability for PE, CMR confirmed pulmonary thromboemboli and right ventricular dysfunction in another 6 cases. Therefore, our results demonstrate the usefulness of a comprehensive MR study for differential diagnosis in selected patients with elevated cTnT but with equivocal findings and non-conclusive diagnoses.

CMR identified 93% of the diagnoses in this CPU patient group. The reason for the minute cTnT rises in two patients (0.03/0.07 ng/l) could not be discovered. In both patients the diagnostic image quality was considerably reduced due to breath-hold incompliance and tachy-arrhythmia. In contrast to previous CT CPU-trials [21], we included also patients with sub-optimal imaging conditions to apply CMR in a clinically realistic setting. Another explanation that we could not find a potential LGE area in the two patients with the slightly elevated cTnT levels could be the employment of our single breath-hold multi-slice-LGE MR sequence holding the disadvantage of a lower image resolution. Therefore, small-sized LGEs could have been overlooked.

Compared to CMR, echocardiography only detected five of eleven AMI patients due to small infarct sizes without wall motion abnormalities. One could argue that perfusion imaging by echocardiography would increase the sensitivity, but even high resolution gadolinium multi-slice perfusion CMR did not reveal noticeable defects. The difference in performance between CMR and echocardiography becomes even more pronounced in patients with PE and peri-/myocarditis, which was the underlying reason in more than 30% of cTnT releases. If PE did not cause RV-dysfunction, echocardiography failed to clarify diagnoses in three patients due to insufficient RV image quality, which is in line with common literature [22]. MR angiography on the other hand enabled visualization of central and peripheral pulmonary thrombi using high-resolution sequences [23]. Also, if peri-myocarditis did not affect wall motion or cause pericardial effusions [22], echocardiography was unable to find the correct diagnosis [24] because of insufficient inflammatory tissue characterisation.

### Feasibility of CMR

All patients were successfully scanned with good image quality and without claustrophobia, with a mean time from first scan to diagnosis of 35 ± 8 min. All CMR scans were conducted between the first and second cTnT measurements (six hours), therefore not causing prolonged patient CPU-stay. CMR could reliably distinguish between hazardous differential diagnoses, which had substantial influence on further diagnostic or therapeutic proceedings. Neither coronary angiography nor echocardiography combined the comprehensive ability of highly reproducible functional as well as and tissue characterisation. Although state-of-the-art CT has superior image resolution for coronary imaging or angiographies, it lacks high-resolution functional and perfusion data so far. Furthermore, inflammatory tissue characterisation cannot be diagnosed appropriately. Thirdly, X-ray and iodine contrast agent administration would have led to a considerable additional radiation exposure in all AMI cases.

### Diagnostic decisions and clinical proceedings after CMR

CMR led to reduced coronary angiography procedures in thirteen cases (6 PE, 5 peri/myocarditis, 2 ESRD), whereas in the 2 ESRD patients the physician decided for non-invasive proceeding because of non-pathological CMR findings. Since CMR predicted all affected coronary AMI territories correctly, it could lead to reduced X-ray exposure in this patient cohort. The concept of CMR implies several essential requirements: 1) short-term clinical access to an CMR-system with a CPU nearby, 2) safe patient monitoring, 3) CPR trained CMR staff, 4) experienced CMR readers and 5) immediate CMR interpretation. Recently published CPU-CMR studies showed a clear clinical benefit in ACS patients [25]. In conclusion, we implemented the Heidelberg CMR-algorithm to show the clinical value in a clinically inconclusive patient cohort showing unclear cTnT elevations. Since this pilot study has shown the CMR feasibility, further prospective studies comparing CMR with different imaging modalities like CT are warranted.

### Limitations

We gave gadolinium in two patients with low GFR (34 ml/min), which requires caution because of the potential induction by some contrast agents of nephrogenic systemic fibrosis (NSF) [26]. Coronary angiography was not conducted in all patients for comparison with CMR and echocardiography. After an unequivocal diagnosis finding, patient management was left at the discretion of the attending cardiologist.

## List of abbreviations

CMR: cardiovascular magnetic resonance; CMP: cardiomyopathy; CPU: chest pain unit; cTnT: cardiac Troponin T; Gadolinium: gadolinium-DTPA; LGE: late gadolinium enhancement; LV/RV: left/right ventricle; NSTEMI/STEMI: (Non-) ST-segment-elevation myocardial infarction; PE: pulmonary embolism; CPR: cardiopulmonary resuscitation.

## Competing interests

The authors declare that they have no competing interests.

## Authors' contributions

HS coordinated the trial together with MMS. HS analysed the data and was responsible for conception and design of the trial and wrote the manuscript. MMS organised the CMR exams and evaluated the data. SL and DL also scanned the patients and analysed some of the data as well as helped drafting the manuscript. HAK and EG revised the manuscript critically for important intellectual content and finally approved the paper. This paper is not under consideration elsewhere and none of the paper's contents have been previously published. All authors have read and approved the manuscript. All authors have no potential conflict of interests.
